# Building a qualitative systems map: applying systems thinking to the commercial determinants of health and industry influence on health policy

**DOI:** 10.1093/eurpub/ckac129.088

**Published:** 2022-10-25

**Authors:** A Bertscher, J Nobles, M Zatonski, A Van Den Akker, S Dance, K Bondy, A Gilmore, M Bloomfield

**Affiliations:** 1 Department of Health, University of Bath, Bath, UK; 2 Bristol Medical School, University of Bristol, Bristol, UK; 3 Department of Social and Policy Sciences, University of Bath, Bath, UK; 4 School of Management, University of Bath, Bath, UK

## Abstract

**Background:**

Unhealthy commodities are major drivers of the global burden of noncommunicable diseases. Commercial actors attempt to influence policy to undermine regulation and existing literature draws attention to the underlying macro-level factors that enable this influence. Public health literature also suggests that industry adapts to regulation and such influence may thus be considered a complex adaptive system. Therefore, this study aimed to build a qualitative systems map to help communicate the complexity of industry influence and develop a tool to facilitate the identification of interventions in follow up research.

**Methods:**

In-person group model grouping workshops were adapted for the online environment. A preliminary qualitative systems map was developed by synthesising two recent studies to facilitate workshop discussions and expedite the mapping process. Twenty-three small group system mapping workshops were conducted with a total of 52 stakeholders, representing researchers, civil society, and public officials from various geographical regions.

**Results:**

The qualitative systems map identifies five pathways through which industry influences policy: a) direct access to public sector decisionmakers; b) creation of confusion and doubt about policy decisions; c) prioritisation of commercial growth; d) industry leveraging the legal and dispute settlement processes; and e) industry leveraging policymaking rules and processes. The pathways contribute to perpetuating macro-level factors that enable industry to deploy practices to influence policy.

**Conclusions:**

A system thinking approach can be applied to industry influence on health policy to depict a complex adaptive system. Interventions need to take into consideration the system's complexity and adaptivity. Further research is needed to test, and improve the systems map and identify interventions to achieve systems change.

